# Comparative Assessment of Real-Time and Offline Short-Lag Spatial Coherence Imaging of Ultrasound Breast Masses

**DOI:** 10.1016/j.ultrasmedbio.2025.01.017

**Published:** 2025-03-11

**Authors:** Nethra Venkatayogi, Arunima Sharma, Emily B. Ambinder, Kelly S. Myers, Eniola T. Oluyemi, Lisa A. Mullen, Muyinatu A. Lediju Bell

**Affiliations:** a Department of Computer Science, Johns Hopkins University, Baltimore, MD, USA; b Department of Electrical & Computer Engineering, Johns Hopkins University, Baltimore, MD, USA; c Department of Radiology & Radiological Science, Johns Hopkins Medicine, Baltimore, MD, USA; d Department of Biomedical Engineering, Johns Hopkins University, Baltimore, MD, USA

**Keywords:** Breast ultrasound, Coherence-based beamforming, Breast cancer, Complicated cysts, GPU programming, Generalized contrast-to-noise ratio

## Abstract

**Objective:**

To perform the first known investigation of differences between real-time and offline B-mode and short-lag spatial coherence (SLSC) images when evaluating fluid or solid content in 60 hypoechoic breast masses.

**Methods:**

Real-time and retrospective (*i.e*., offline) reader studies were conducted with three board-certified breast radiologists, followed by objective, reader-independent discrimination using generalized contrast-to-noise ratio (gCNR).

**Results:**

The content of 12 fluid, solid and mixed (*i.e*., containing fluid and solid components) masses were uncertain when reading real-time B-mode images. With real-time and offline SLSC images, 15 and 5, respectively, aggregated solid and mixed masses (and no fluid masses) were uncertain. Therefore, with real-time SLSC imaging, uncertainty about solid masses increased relative to offline SLSC imaging, while uncertainty about fluid masses decreased relative to real-time B-mode imaging. When assessing real-time SLSC reader results, 100% (11/11) of solid masses with uncertain content were correctly classified with a gCNR<0.73 threshold applied to real-time SLSC images. The areas under receiver operator characteristic curves characterizing gCNR as an objective metric to discriminate complicated cysts from solid masses were 0.963 and 0.998 with real-time and offline SLSC images, respectively, which are both considered excellent for diagnostic testing.

**Conclusion:**

Results are promising to support real-time SLSC imaging and gCNR application to real-time SLSC images to enhance sensitivity and specificity, reduce reader variability, and mitigate uncertainty about fluid or solid content, particularly when distinguishing complicated cysts (which are benign) from hypoechoic solid masses (which could be cancerous).

## Introduction

Ultrasound is an important supplement to mammography, which has long been the standard screening method for breast cancer [1]. However, the effectiveness of mammography is compromised in women with dense breast tissue and ultrasound also has limitations [2–4]. Although ultrasound helps to visualize lesions in mammographically dense breasts, its high false-positive rates lead to unnecessary biopsies and follow-up procedures for benign fluid-filled cysts [4,5]. In the American College of Radiology Imaging Network 6666 trial, more false positives were seen with ultrasound with increased breast density (e.g., 10.2% false positives with <25% density, increasing to 14.4% false positives with >80% breast density) [5].

False positives with ultrasound B-mode imaging are often caused by image artifacts such as acoustic clutter, which is partially responsible for similarities in the appearance of complicated cysts and hypoechoic solid masses [6,7]. Acoustic clutter is caused by multi-path acoustic interactions, phase aberration and off-axis scattering [6], and is particularly problematic in acoustically heterogeneous breast tissue.

Harmonic imaging reduces acoustic clutter by leveraging the non-linear propagation of acoustic waves in biological tissues to minimize acoustic reverberations, suppress side and grating lobes and produce narrower beamwidths, offering additional advantages over traditional fundamental B-mode imaging, such as improved organ visualization and enhanced lateral resolution [8–10]. In breast imaging, harmonic imaging improves lesion characterization and margin assessment, particularly when differentiating fluid-filled from solid masses [9,10]. However, the effectiveness of harmonic imaging can be influenced by tissue composition, with its benefits diminished in dense (as opposed to fatty) tissue [9,11]. Additional challenges associated with harmonic imaging in dense breast tissue include increased shadowing and reduced penetration depths at higher frequencies [3].

Alternatives to harmonic imaging include shear wave imaging [12–14], strain imaging [15–18], quantitative ultrasound [19–24] and ultrasound tomography [25,26]. However, these approaches tend to minimize many of the historical benefits of ultrasound imaging. For example, the accuracy of shear wave and strain imaging depends on loading conditions introduced by the probe pressure to maintain acoustic coupling, and shear wave imaging additionally requires system power specifications that are capable of large acoustic outputs [27–30]. Quantitative ultrasound requires careful calibration methods [31], and ultrasound tomography requires dedicated hardware with high channel counts and with less portability than traditional ultrasound systems [32].

Coherence-based imaging, particularly short-lag spatial coherence (SLSC) imaging [33], is promising for ultrasound imaging of dense breasts [34,35]. This technique is known to reduce acoustic clutter while maintaining many of the traditional ultrasound imaging benefits. For example, SLSC imaging does not rely on loading conditions, necessitate custom calibration phantoms nor require custom hardware with reduced portability. In addition, the sensitivity and specificity of coherence-based imaging can outperform clinical elastography techniques [36].

SLSC images are based on local measurements of the spatial coherence of backscattered echoes rather than the amplitude of backscattered echoes that is the fundamental basis for other ultrasound image formation methods (*e.g*., B-mode, harmonic imaging, shear wave imaging, quantitative ultrasound, ultrasound tomography). Initially demonstrated as a technique to reduce acoustic clutter and improve ultrasound image quality in clinical applications such as thyroid [33], cardiac [37,38], fetal [39,40], liver [41] and needle [42] imaging, SLSC has clear potential to differentiate hypoechoic solid from fluid breast masses [34–43]. This differentiation is possible because solid masses have similar spatial coherence relative to surrounding tissue, whereas fluid masses have lower spatial coherence, which assists with complicated cyst distinction [34,35].

In a reader study with five board-certified breast radiologists, a modification to SLSC imaging termed robust short-lag spatial coherence (R-SLSC) [44] decreased uncertainty regarding fluid-filled mass content from 47.5% to 15.8%, which decreased the percentage of fluid-filled masses recommended for biopsy from 43.3% to 13.3% [36]. However, there was inter-reader variability with moderate agreement among the five readers when assessing the sensitivity of detecting fluid-filled masses. To reduce subjectivity, objective metrics such as contrast, contrast difference, lag-one coherence, coherence length and generalized contrast-to-noise ratio (gCNR) were investigated [34–36,43]. Most recently, gCNR with a pre-defined threshold of 0.73 produced similar sensitivity and greater specificity compared to that of the previously prevailing lag-one coherence metric, when applied to SLSC and R-SLSC images [35]. SLSC imaging was also superior to harmonic B-mode imaging when making this objective distinction between fluid and solid mass contents [35]. Therefore, gCNR applied to SLSC images has the greatest potential to objectively improve the distinction between fluid and solid masses [35].

The coherence-based breast imaging results summarized above [34–43] were obtained through offline analyses conducted outside of the patient examination room. With the development of real-time GPU SLSC implementations [45–48] and deep learning solutions [49] clinical deployment is feasible, with R-SLSC imaging requiring more computational steps to implement in comparison to SLSC imaging. There are also known image quality trade-offs between real-time and offline SLSC implementations due to the signal-processing shortcuts required for GPU implementations [45–49]. We are primarily interested in evaluating real-time SLSC approaches, with the understanding that findings can potentially be translated to more advanced SLSC techniques with more computing resources in future implementations.

A preliminary study from our group [48] deployed real-time SLSC imaging on 47 hypoechoic breast masses, resulting in three radiologists correctly classifying four complicated cysts as Breast Imaging-Reporting and Data System (BI-RADS) category 2 in the patient examination room, whereas one was uncertain with B-mode imaging and two were recommended for follow-up (BI-RADS 3) or biopsy (BI-RADS 4) with B-mode imaging. Although we anticipate greater reader variability [50] with more masses included, the previously promising results obtained with the objective gCNR metric applied to SLSC images created after patient exams were completed (i.e., offline) [35] are expected to successfully translate to similarly positive outcomes when gCNR is applied to SLSC image screenshots acquired during real-time acquisitions. This improvement is expected despite known differences introduced by the real-time GPU signal-processing shortcuts.

The purpose of the study herein is to evaluate potential differences between gCNR results obtained with real-time and offline SLSC images with respect to reader classifications of hypoechoic masses. A secondary objective is to determine the extent to which real-time SLSC imaging and gCNR applied to either real-time B-mode or SLSC images can assist with uncertain B-mode evaluations of mass content. To achieve these objectives, we conducted a reader study comparing offline and real-time SLSC and B-mode images, and subsequently evaluated the performance of gCNR as an objective metric when applied to real-time screenshots and images that were beamformed offline.

## Methods

### Proposed process

**Figure 1.**
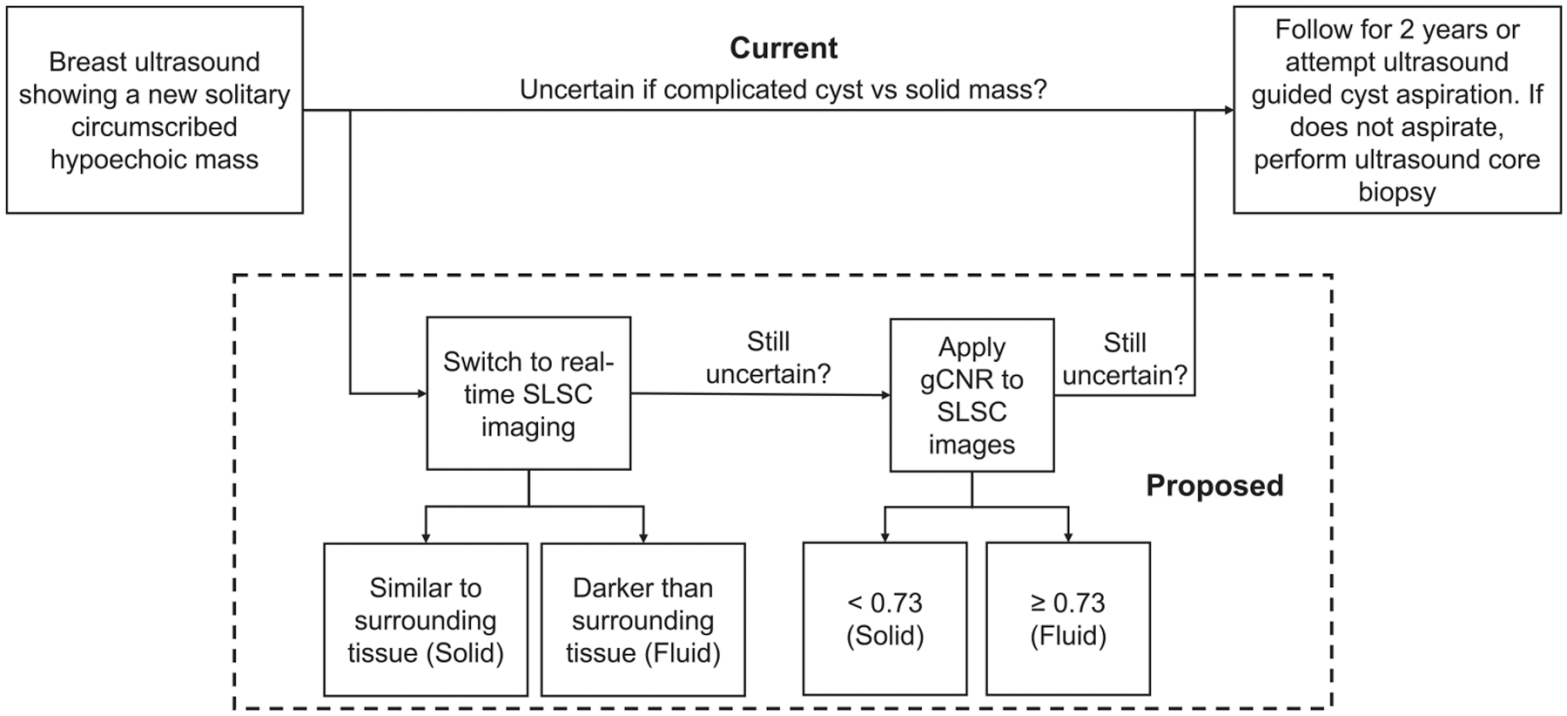
Comparison of current workflow with our proposed clinical workflow to implement short-lag spatial coherence (SLSC) and generalized contrast-to-noise ratio (gCNR), which applies when a new mass is identified through callback a from diagnostic or screening mammogram or through a screening breast ultrasound of dense breast tissue.

We contrast the current real-time process in cases of uncertainty with our proposed process, which incorporates SLSC and gCNR, as summarized in Figure 1. First, we acknowledge that traditional B-mode breast ultrasound imaging is expected to be sufficient in some cases (*e.g*., simple cysts [51,52] or malignant masses with highly suspicious imaging features such as irregular shape and spiculated margins [53–55]). With our proposed process, viewing SLSC images may be sufficient to determine solid or fluid content in cases of traditional uncertainty (*e.g*., complicated cysts) [34,35,56,57]. If a reader remains uncertain about fluid or solid mass contents after viewing SLSC images, we propose a follow-up calculation of the gCNR of the SLSC images. With the outcome of this gCNR calculation provided, it is possible that there may be some residual uncertainty (*e.g*., due to borderline cases near the chosen gCNR threshold, lack of trust of new technology, auxiliary image features of concern, the clinical history of the patient, etc.). In these cases of uncertainty, it would be reasonable to proceed with the typical protocol that is already familiar to the radiologist (see last step in Fig. 1).

### Study population

**Figure 2.**
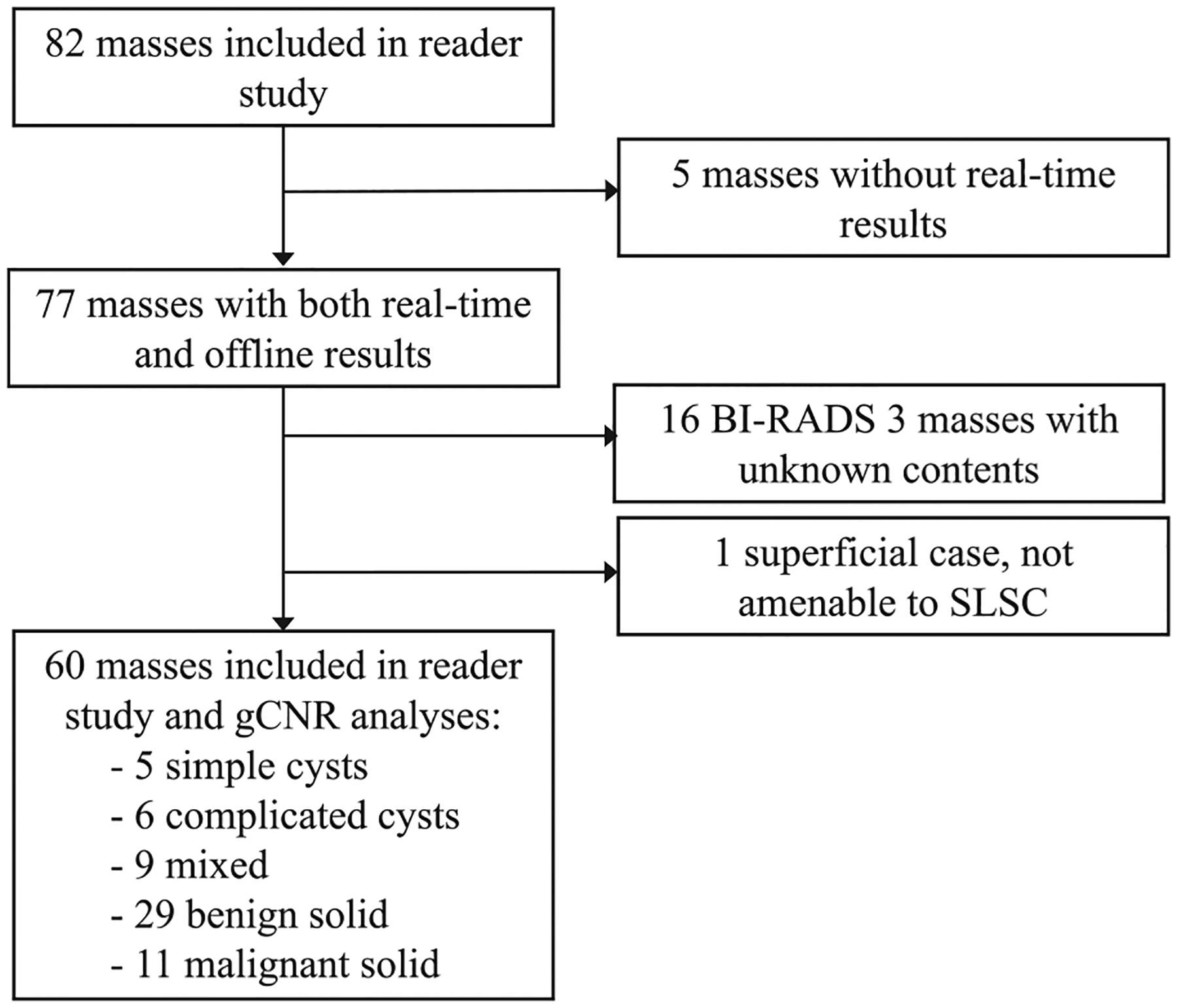
Flowchart summarizing the inclusion and exclusion of breast masses when performing reader study analyses and applying gCNR as an objective discriminator of mass content.

Patients who met the following four inclusion criteria were recruited: (i) adult females (>18 y), (ii) English speakers, (ii) not pregnant and (iv) scheduled for ultrasound-guided core-needle biopsy, aspiration or follow-up of at least one breast mass. A total of 44 patients (mean ± standard deviation age: 51 ± 17 y) with 82 masses in total were enrolled after obtaining written informed consent. Sixty of the 82 masses were eligible for analysis, including five simple cysts present in addition to the masses scheduled for aspiration or biopsy. As documented in Figure 2, masses were excluded from analysis based on pathological, imaging or physical characteristics (*i.e*., BI-RADS 3 masses have no pathological results, real-time SLSC imaging was required, shallow masses are not suitable for SLSC imaging [35]). When the differential diagnosis included a cyst, ultrasound-guided aspiration was initially attempted, resulting in a complicated cyst classification upon successful aspiration. Simple cysts were classified without aspiration based on clinical ultrasound B-mode features. Otherwise, the results of each core-needle biopsy served as the ground truth for mass classification. This study was approved by the Johns Hopkins Institutional Review Board (protocol no. IRB00127110).

### Data acquisition and processing

Each patient was imaged by a board-certified breast radiologist co-author (E.A., K.M. or E.O.). An Alpinion ECUBE12R research ultrasound scanner (Alpinion, Seoul, South Korea) connected to a 128-element, L8–17 probe with 64 receive elements, 12.5 MHz center frequency and 40 MHz sampling frequency was employed to acquire raw ultrasound radiofrequency (RF) channel data with 256 receive scan lines per image. The focus of transmitted beams was located within 0–1 cm of the mass center, as selected by the radiologist co-author performing the scan. Raw RF ultrasound data of two orthogonal views per mass were acquired and saved for post-procedure processing. In addition, clinical screenshots of real-time B-mode images (processed using proprietary information) and real-time SLSC images (processed using the details reported in [47,48], with additional proprietary processing information added to the real-time display), each located within the same buffer as the RF data, were concurrently acquired. These data were acquired from January 24, 2023 through October 5, 2023.

To process acquired RF data offline and create offline B-mode images, a standard delay-and-sum beamformer was implemented. To form offline SLSC images [33,34,36,58], delays were first applied to the acquired RF data to account for time-of-arrival differences. Then, data within correlation kernel lengths equal to one wavelength were cross-correlated to create coherence functions, $\overset{ˆ}{R}$, for each lateral and axial position in the image [33]:

(1) $$\overset{ˆ}{R}(m)=\frac{1}{N-m}\overset{N-m}{\underset{i=1}{\sum }}\frac{\sum_{n=n_{1}}^{n_{2}}s_{i}(n)s_{i+m}(n)}{\sqrt{\sum_{n=n_{1}}^{n_{2}}s_{i}^{2}(n)\sum_{n=n_{1}}^{n_{2}}s_{i+m}^{2}(n)}}$$

where m is the spatial lag (expressed as the number of element separations), N is the number of transducer elements, $s_{i}(n)$ is the time-delayed, zero-mean signal received at element i from depth n, and the axial correlation kernel, k, spans depths $n_{1}$ to $n_{2}$, centered on depth n. An SLSC image pixel was created by summing the resulting spatial coherence function up to a short-lag value, M:

(2) $$R_{sl}=\int_{1}^{M}\overset{ˆ}{R}(m)\text{dm}\approx \overset{M}{\underset{m=1}{\sum }}\overset{ˆ}{R}[m]$$

Eqns (1) and (2) were repeated in succession for each lateral and axial SLSC pixel location. Negative SLSC pixels were set to zero (based on the rationale that these small negative pixels adversely affect image quality and contrast measurements [44]).

Contrary to the offline processing method for SLSC images, eqns (1) and (2) were manipulated to fully utilize the benefits of the GPU embedded in the ultrasound system and form real-time SLSC images. In particular, as described in previous work [47,48], the GPU SLSC implementation first computes individual correlation $\left(C_{i,i}\right)$ and autocorrelation ($C_{i,i}$ and $C_{i,j}$) terms for scanline x, axial sample z, and elements i and j separated by m, given by the following equations:

(3) $$C_{i,j}(z,x,m)=\overset{N_{i}-m}{\underset{i=1}{\sum }}s_{i}(z,x)s_{i+m}(z,x{)}^{*},$$

(4) $$C_{i,i}(z,x,m)=\overset{N_{i}-m}{\underset{i=1}{\sum }}{\left|s_{i}(z,x)\right|}^{2}$$

(5) $$C_{j,j}(z,x,m)=\overset{N_{i}-m}{\underset{i=1}{\sum }}{\left|s_{i+m}(z,x)\right|}^{2}$$

where * denotes the complex conjugate. $C_{i,j},C_{i,i}$, and $C_{j,j}$ were stored in the device global memory and then compounded across k, up to M, as follows:

(6) $$\text{SLSC}(z,x)=\overset{M}{\underset{m=1}{\sum }}\frac{\sum_{\overset{ˆ}{z}\in k}C_{i,j}(\overset{ˆ}{z},x,m)}{\sqrt{\sum_{\overset{ˆ}{z}\in k}C_{i,i}(\overset{ˆ}{z},x,m)\sum_{\overset{ˆ}{z}\in k}C_{j,j}(\overset{ˆ}{z},x,m)}}$$

Considering the differences relative to the offline data-processing method (coupled with the proprietary display differences noted above), differences between real-time and offline image appearances are possible. Hence, the primary objective of the presented work was to compare the impact of such differences.

### Real-time reader study

In total, 77 masses were reviewed independently in real time (*i.e*., in the patient examination room) by the radiologist performing the ultrasound scan. Although each radiologist read 20−33 of the 77 masses included in the real-time reader study (corresponding to cases seen by them), only 60 masses were included in the analysis of results, as shown in Figure 2. In particular, Readers 1, 2 and 3 read 18 masses (2 simple cysts, 1 complicated cyst, 12 benign solid, 1 malignant solid and 2 mixed), 22 masses (3 simple cysts, 1 complicated cysts, 10 benign solid, 6 malignant solid and 2 mixed) and 20 masses (4 complicated cysts, 7 benign solid, 4 malignant solid and 5 mixed), respectively. Each radiologist had experience beyond residency ranging from 6 to 11 years on the first day of data acquisition.

To perform real-time readings, the radiologists classified mass content (*i.e*., solid, fluid, mixed or uncertain) and provided a clinical diagnosis (*i.e*., the BI-RADS category) based on orthogonal views of each mass, obtained with the clinical grade B-mode images provided by the in-built ultrasound beamformer. The ultrasound scanner was then switched to its real-time SLSC imaging mode and the same radiologist scanned the same mass to obtain similar orthogonal views and provide classifications (*i.e*., content followed by BI-RADS category) using the additional real-time SLSC information.

### Offline reader study

To investigate potential interpretability differences when reading real-time or offline images and to assess the ability of individual readers to return to the same result, a series of retrospective (or offline) readings were performed after the real-time readings were completed. Images from 82 masses (including the 77 masses acquired during the real-time study) were randomized and presented to the same three readers using an updated version of the graphical user interface illustrated in [36]. Updates included an option to toggle between the two orthogonal orientations of each mass, a dropdown menu to specify the type of mixed masses (*e.g*., complex cystic and solid mass, cluster of cysts, fat necrosis, clinical abscess, or an option to type an alternative description) and a text box presenting clinical notes containing a concise patient summary and history.

To avoid reader bias based on clinical notes and ensure uniform inclusion of information, the clinical history of each patient was reviewed and summarized by a radiologist who did not perform the scan. The reviewing radiologist summarized the following details as the case history for each patient: age, reason for examination (callback, palpable, follow-up, screening, concern for infection), whether the ultrasound mass had a physical examination or mammographic correlate (vs. an incidental finding), stability and details of any priors available for the finding.

For each mass, the readers were presented with two tasks (*i.e*., Task 1 followed by Task 2) prior to advancing to the next mass. In Task 1, only B-mode images were presented, including both the clinical screenshot and the reconstructed B-mode image formed from the saved RF data. Both of these B-mode images were presented to avoid bias from the poorer quality B-mode images created from saved RF data, which were processed without the non-linear filters that are present in most clinical systems. With only B-mode images, readers selected the content—(1) solid, (2) fluid, (3) mixed or (4) uncertain—and the BI-RADS category—(2) benign finding, (3) probably benign, (4) suspicious abnormality and (5) highly suggestive of malignancy—of each mass. In Task 2, readers were additionally presented with SLSC images of the same mass and selected the content and BI-RADS category. In addition, a duplex display that overlaid the SLSC image on the B-mode image [59] was available if desired.

For Tasks 1 and 2, the readers had full control over tunable parameters, including dynamic range and short-lag value (*i.e*., M in eqn [2]). The responses of each radiologist were saved for comparison with real-time assessments and to provide statistical analyses summarizing intra-reader agreement. All readings were performed in a dark room with the same 23.8-inch monitor. Readers had the autonomy to manage their study pace and breaks to prevent reader fatigue. This offline study was designed to be as similar as possible to the real-time study. Although each radiologist read 24−33 of the 82 masses included in the offline reader study (corresponding to cases seen by them), only 60 cases were included in the analysis of results, as summarized in Figure 2.

### Reader-independent assessment

To perform the quantitative reader-independent assessment of mass content in B-mode and SLSC images, regions of interest (ROIs) within the 60 masses summarized in Figure 2 and surrounding tissue were manually selected from offline B-mode images. The mass ROI was an elliptical area within the mass. The tissue ROI was the same shape, size and depth as the mass ROI, with its nearest edge located at a lateral distance of 0.9–8.2 mm from the nearest edge of the mass ROI. However, if a mass spanned most of the lateral field of view, the tissue ROI location was modified to a depth <5 mm from the nearest edge of the mass ROI. Per mass, the same mass and tissue ROIs were implemented when calculating the gCNR of B-mode and SLSC images as follows [60,61]:

(7) $$\text{gCNR}=1-\overset{N}{\underset{j=1}{\sum }}\text{min}\left\{h_{\text{mass}}\left(x_{j}\right),h_{\text{tissue}}\left(x_{j}\right)\right\}$$

where N bins centered at $\left\{x_{1},x_{2}\ldots x_{N}\right\}$ were defined to derive histograms $h_{\text{mass}}$ and $h_{\text{tissue}}$ of the beamformed signals (after envelope detection for B-mode images, prior to normalization and log compression for B-mode and SLSC images) within the mass and tissue ROIs, respectively, and j is the index of the bin. The gCNR of SLSC images was computed with M=7, corresponding to 10% of the receive aperture, as implemented in [35]. To calculate gCNR using clinical screenshots, images were first converted to grayscale and double precision, and the same ROIs selected from the offline B-mode images were employed to mitigate potential variations between the intended real-time and offline gCNR comparisons. The gCNR measurements calculated from each clinical screenshot corresponding to the offline images are considered to be pseudo-real-time results, representing the gCNR values that would otherwise be obtained from the real-time images.

### Statistical analyses

To determine intra-reader agreement between the real-time and offline results, Fleiss’ κ was calculated [62], with values <0.0, 0−0.20, 0.21−0.40, 0.41−0.60, 0.61−0.80 and >0.81 interpreted as poor, slight, fair, moderate and substantial agreement, respectively, in accordance with [63]. Mixed masses were excluded because individual reader interpretation can be variable as these masses contain cystic and solid content.

The sensitivity and specificity of complicated cyst detection were measured as the fraction of complicated cysts correctly identified as fluid and the fraction of solid masses correctly identified as solid, respectively:

(8) $$\text{Sensitivity}=\frac{\text{TP}}{\text{TP}+\text{FN}}$$

(9) $$\text{Specificity}=\frac{\text{TN}}{\text{TN}+\text{FP}}$$

where true positive (TP) and false negative (FN) are defined as complicated cysts with ground truth content classification as fluid and not fluid, respectively. Conversely, true negative (TN) and false positive (FP) are defined as solid masses with ground truth content classification as solid and not solid, respectively. Masses with a ground truth classified as mixed were omitted from this analysis, considering that they contain a mixture of TP and TN. Simple cysts were also excluded, considering that traditional B-mode images are typically sufficient to identify features of anechoic simple cysts [51,52].

The sensitivity and specificity of the objective, reader-independent gCNR metric for complicated cyst detection were also calculated using eqns (8) and (9) for gCNR threshold values ranging from 0 to 1 (with simple cysts and mixed masses excluded for the same reasons summarized above). More specifically, B-mode and SLSC images of a complicated cyst were considered TP or FN if the gCNR value of the image was above or below a specific threshold, respectively. Conversely, B-mode and SLSC images of a solid mass were considered TN or FP if the gCNR value of the image was below or above the threshold, respectively. The gCNR threshold for complicated cyst detection was varied from 0 to 1 in increments of 0.01. Sensitivity was plotted against 1-specificity for each threshold to form the receiver operating characteristic (ROC) curve. The area under the ROC curve (AUC) was estimated using trapezoidal numerical integration. This process was implemented for both the offline gCNR values and the pseudo-real-time gCNR results, resulting in offline and real-time gCNR performance details that enabled direct comparisons of these two approaches.

## Results

### Intra-reader variability

**Figure 3.**
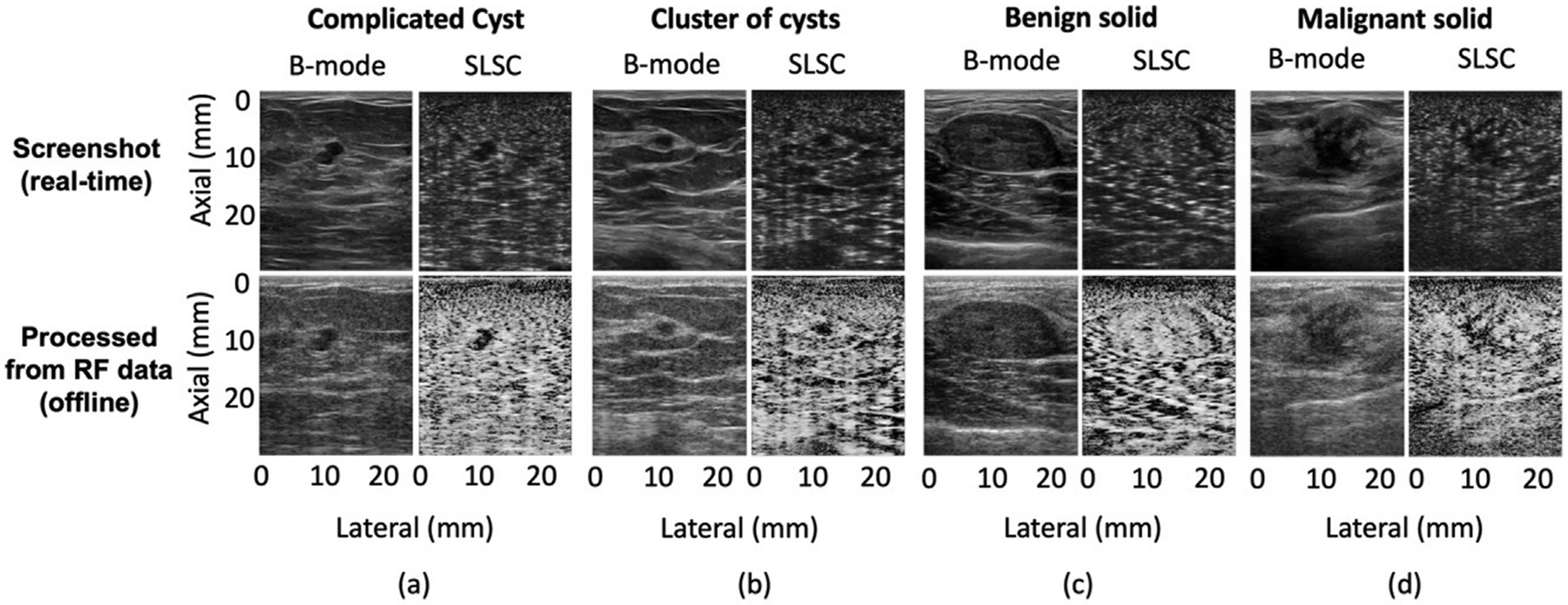
Real-time (*top row*) and offline (*bottom row*) B-mode and SLSC images of an example each of (a) complicated cyst, (b) cluster of cysts, (c) benign solid mass and (d) malignant solid mass. All images are displayed with 60 dB dynamic range. Reproduced from [48].

Figure 3 shows screenshots of real-time and offline B-mode and SLSC images of representative hypoechoic breast masses. The spatial coherence of fluid-filled (*i.e*., complicated cyst) and mixed (*i.e*., cluster of cysts) masses is lower than that of the surrounding tissue, resulting in a darker appearance of the masses relative to the surrounding tissue in both real-time and offline SLSC images. In contrast, the benign and malignant solid masses and the surrounding tissue have similar spatial coherence in both real-time and offline SLSC images. Notable differences between real-time and offline images (*e.g*., signal amplitude, clutter, image texture) can be attributed to proprietary, in-built, non-linear filters applied prior to taking the screenshots, as well as variations between offline and GPU SLSC beamformers. These examples represent variations among the images presented in the real-time and offline reader studies.

**Figure 4.**
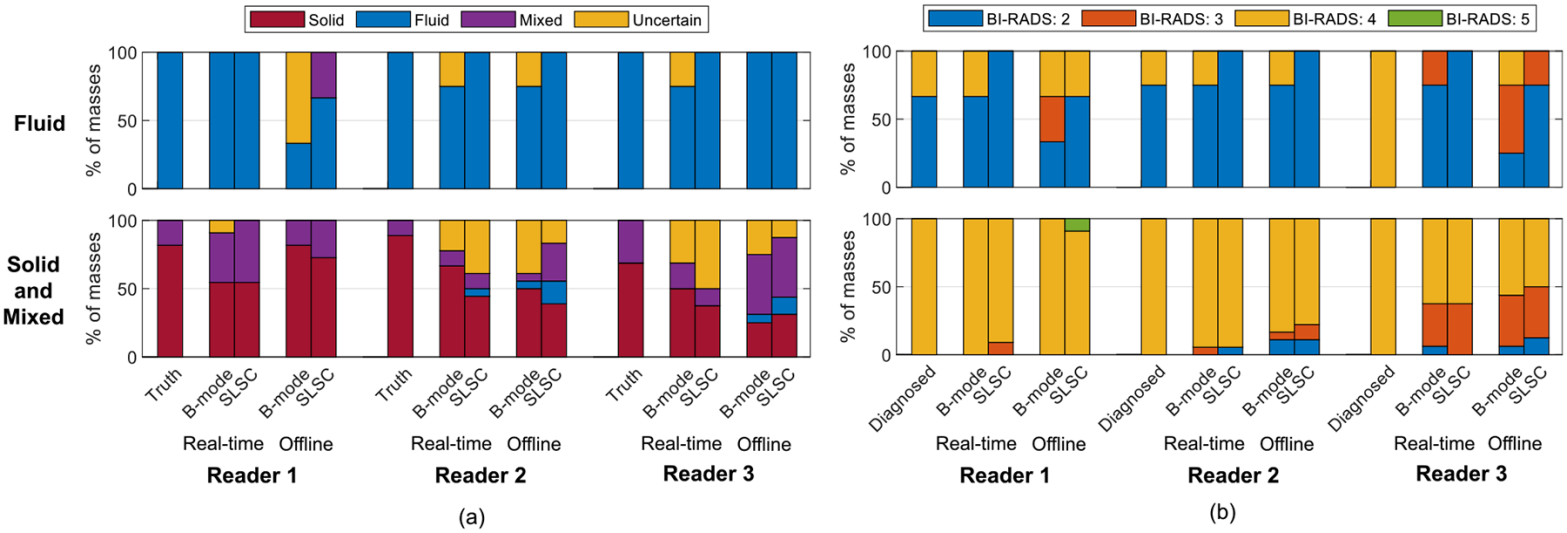
(a) Content and (b) BI-RADS classification by Readers 1, 2 and 3 during real-time and corresponding offline assessments.

Figure 4 summarizes the reader assessment results. The ground truth is followed by pairs of assessments obtained in real-time and offline per reader. The left bar in each pair indicates the reader decision using only B-mode images, and the right bar indicates the reader decision when considering B-mode alongside SLSC images. Each color represents the percentage of the masses classified in each category. SLSC imaging consistently reduced the uncertainty of determining fluid mass content (Fig. 4a) and the percentage of BI-RADS 3 or 4 assessments of fluid masses (Fig. 4b) in real-time and offline, per reader. In particular, the uncertainty of fluid-filled mass content was reduced from 2/11 (18%) with real-time B-mode imaging to 0/11 (0%) when including real-time SLSC imaging, which decreased the number of fluid-filled masses that would have been recommended for biopsy or follow-up from 3/11 (27%) to 0/11 (0%). One of the fluid masses was recommended for biopsy due to its margins, which were thought to be suspicious and led to uncertainty about the cystic nature of the finding. With offline imaging, the uncertainty of fluid-filled mass content was reduced from 3/11 (27%) with B-mode to 0/11 (0%) when including SLSC, which decreased the number of fluid-filled masses that would have been recommended for biopsy or follow-up from 6/11 (54%) to 2/11 (18%). The largest percentage of uncertainty existed when readers assessed solid and mixed masses with real-time SLSC images. In particular, 10/49 (20%), 12/49 (24%), 15/49 (31%) and 5/49 (10%) solid and mixed masses were uncertain with real-time B-mode, offline B-mode, real-time SLSC and offline SLSC images, respectively.

When performing content classification and summarizing the intra-reader agreement between real-time and offline assessments, fair agreement was observed with B-mode images (*i.e*., κ=0.36, 0.38 and 0.34 for Readers 1, 2 and 3, respectively). These Fleiss’ κ-values increased to 0.89, 0.45 and 0.54, respectively, after including SLSC images, demonstrating moderate (Readers 2 and 3) to perfect (Reader 1) agreement [63]. When performing BI-RADS classification with B-mode images, there was moderate (Reader 3, κ=0.53) to substantial (Readers 1 and 2, κ=0.72 and 0.62, respectively) agreement. This agreement decreased to moderate for Reader 1 (κ=0.60) and increased to substantial for Readers 2 and 3 (κ=0.68 and 0.79, respectively) after including SLSC images.

### gCNR compared with subjective reader assessment

**Figure 5.**
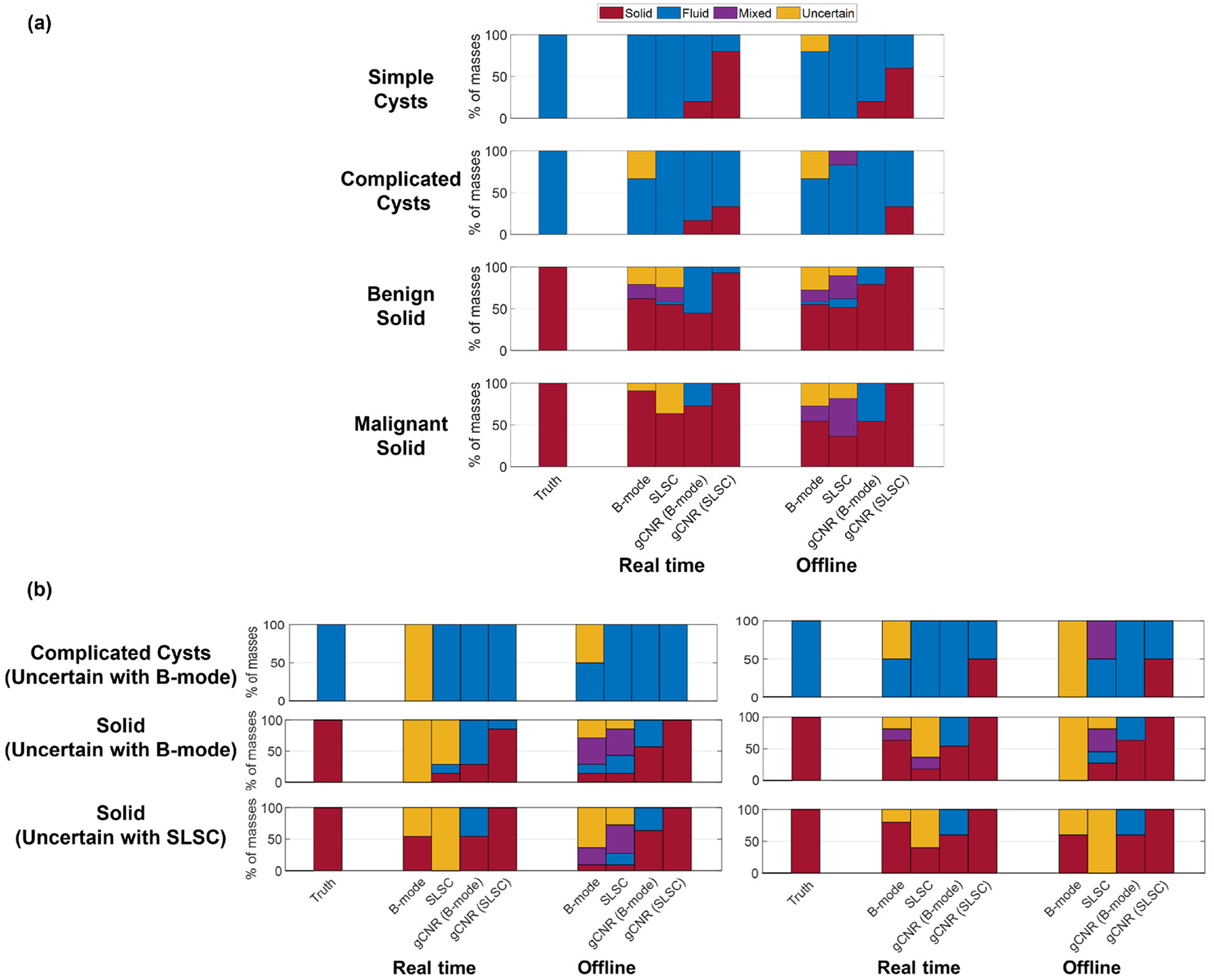
(a) Aggregated content classification of masses assessed by three radiologists during real-time and corresponding offline assessments and with a gCNR threshold of 0.73. (b) Corresponding subsets of masses that experienced reader uncertainty in two different groups of two complicated cysts (uncertain with real-time and offline B-mode images), one group of seven solid masses (uncertain with real-time B-mode images), one group of five solid masses (uncertain with offline SLSC images), and two different groups of 11 solid masses (uncertain with offline B-mode images and real-time SLSC images).

Figure 5a shows the aggregated mass contents classified by the three radiologists along with the corresponding gCNR classifications in real-time and offline images, excluding nine mixed masses. The additional, objective gCNR discriminator was applied to clinical screenshots (*i.e*., the pseudo-real-time result) and offline B-mode and SLSC images using the previously determined threshold of 0.73 to distinguish fluid from solid masses [64]. Although this gCNR threshold misclassified 4/5 (80%) simple cysts when applied to real-time SLSC images, this misclassification is not considered problematic because traditional B-mode images are typically sufficient to identify features of anechoic simple cysts [51,52]. Notably, 5/5 (100%) simple cysts in Figure 5a were correctly characterized by radiologists with real-time B-mode images and with real-time and offline SLSC images. There is dissatisfying uncertainty when reading B-mode images to distinguish complicated cysts from solid masses, which appears to be mitigated with SLSC imaging and/or gCNR.

Figure 5b shows the results of only the aggregated complicated cysts and solid masses that were marked uncertain with either B-mode or SLSC images during real-time or offline assessments. In summary, 8/9 (89%) complicated cysts and solid masses that were uncertain with real-time B-mode images were correctly classified with gCNR applied to real-time SLSC images, whereas 12/13 (92%) complicated cysts and solid masses that were uncertain with offline B-mode images were correctly classified with gCNR applied to offline SLSC images. Although there was no uncertainty with SLSC imaging of complicated cysts, as shown in Figure 5a, 11/11 (100%) solid masses classified as uncertain with real-time SLSC images and 5/5 (100%) solid masses classified as uncertain with offline SLSC images were correctly classified as solid with gCNR applied to real-time or offline SLSC images. Overall, gCNR applied to real-time and offline SLSC images increased the confidence of distinguishing complicated cysts from solid masses by 89−100%. There are also isolated cases of complicated cyst uncertainty that benefit from applying gCNR to B-mode images.

**Figure 6.**
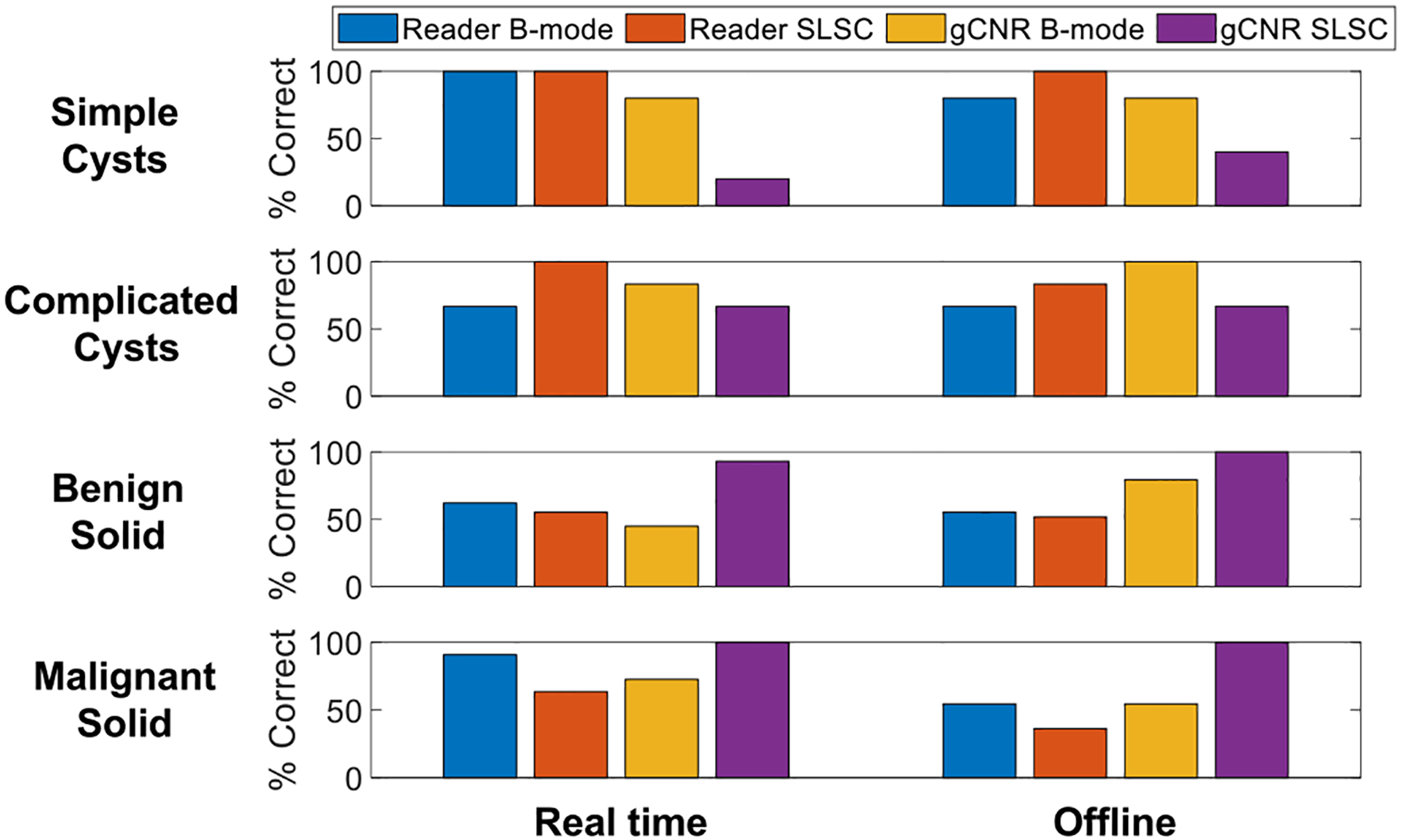
Classification accuracy of real-time and offline B-mode and SLSC, including reader results and gCNR results obtained with the 0.73 threshold, per mass category.

Figure 6 summarizes the percentage of correct classifications for the 5 simple cysts, 6 complicated cysts, 29 benign solid masses and 11 malignant solid masses included in our study (as opposed to only summarizing correct classifications for the subset of masses that were uncertain). Results are stratified by mass category and B-mode or SLSC reader or gCNR outcomes, with side-by-side comparisons of real-time and offline assessments. Focusing on the real-time results, as noted above, simple cysts are best classified when reading real-time B-mode (or SLSC) images (*i.e*., 100% correct), complicated cysts are best classified with real-time SLSC image readings (*i.e*., 100% correct), and benign and malignant solid masses are best classified with gCNR applied to SLSC images (*i.e*., 93% and 100% correct, respectively). The offline results provide similar outcomes, with differences possibly due to variations between real-time and offline images observed in Figure 3.

**Figure 7.**
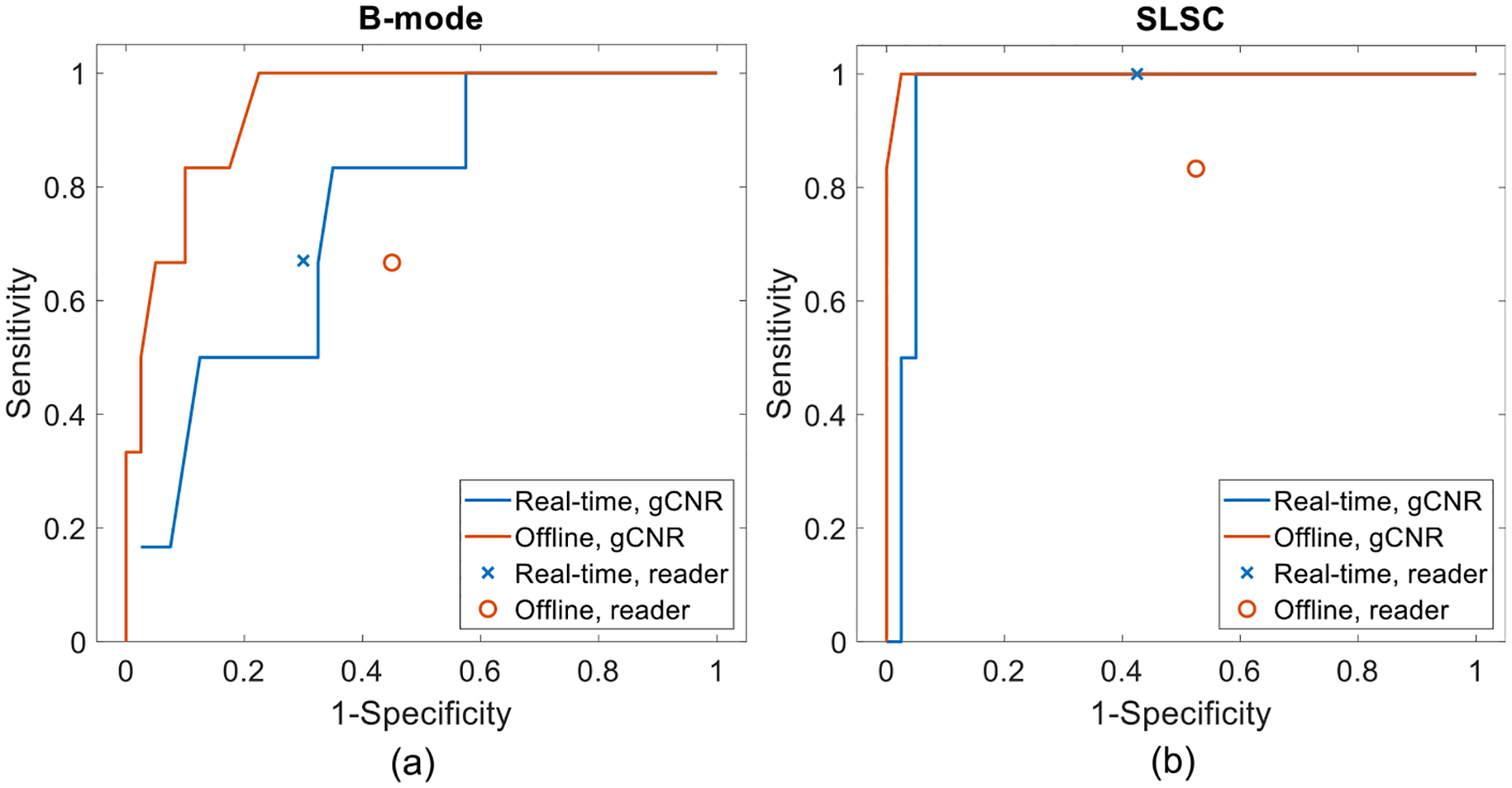
Receiver operating characteristic curves demonstrating the performance of gCNR as an objective metric to distinguish complicated cysts from solid masses, using real-time and offline (a) B-mode images and (b) SLSC images, relative to the aggregated performance of three readers.

Figure 7 shows ROC curves when distinguishing complicated cysts from solid masses with gCNR. In Figure 7a, the AUCs with gCNR applied to real-time and offline B-mode images are 0.756 and 0.940, respectively. In Figure 7b, the AUCs with gCNR applied to real-time and offline SLSC images are 0.963 and 0.998, respectively, which is considered excellent (*i.e*., ≥ 0.9) in diagnostic testing [65,66]. The sensitivity and 1-specificity of the three readers are plotted for comparison.

Table 1 compares reader sensitivity and specificity to that achieved with the previously determined gCNR = 0.73 threshold [64] and with the gCNR threshold yielding the closest point to (0,1) on the ROC curve (*i.e*., gCNR = 0.62), highlighting the performance of readers relative to gCNR. Reader under-performance with respect to solid masses (*i.e*., poor specificity) was primarily due to uncertainty, which increases false positives.

## Discussion

This paper is the first to evaluate the effectiveness of real-time SLSC imaging, relative to historical offline SLSC implementations, when differentiating fluid from solid masses. The results are evaluated with respect to reader assessments and gCNR as an objective metric, with four key insights. First, radiologist readings can produce variable results relative to the ground truth with both real-time and offline methods (Fig. 4), and this variability was consistent per reader (*e.g*., Fleiss’ κ indicated moderate-to-perfect agreement between real-time and offline SLSC readings per radiologist). Second, the percentage of uncertain fluid masses in the patient examination room decreased from 18% with real-time readings of B-mode images to 0% with real-time readings of SLSC images. Third, 89−100% of complicated cysts and solid masses that were initially uncertain when reading B-mode or SLSC images were correctly classified when gCNR was applied to SLSC images (Fig. 5). Finally, there are minimal differences between gCNR results obtained with real-time and offline SLSC images, as demonstrated with the majority of gCNR thresholds in Figure 7b. Based on these insights, the proposed workflow presented in Figure 1 is expected to benefit clinical outcomes, leading to reduced uncertainty, fewer masses being unnecessarily followed for 2 years and fewer recommendations for invasive diagnostic procedures (*e.g*., biopsy, aspiration).

In a previous study, coherence-based beamforming increased the diagnostic certainty of distinguishing fluid from solid masses and decreased the percentage of fluid-filled masses that would have been recommended for biopsy [36]. The number of masses included, the sensitivity and specificity definitions, and the SLSC approach presented herein differed from previous approaches [35,36], which contributes to differences in absolute percentages, sensitivity and specificity. We also observed greater uncertainty when assessing solid masses, likely due to dark-region artifacts, which are more prominent in SLSC (rather than the previously assessed R-SLSC) images [35,36,67]. Two preliminary reports introduced real-time SLSC imaging as a promising method to reduce reader uncertainty with B-mode images [48] and gCNR as a reader-independent metric to distinguish fluid from solid masses [64], which are both supported and strengthened by the new contributions presented herein.

One logistical challenge with implementing gCNR is that background and mass ROIs need to be selected. As there are typically two orthogonal orientations per mass (*i.e*., radial and anti-radial views), varying the selected ROI per orientation might alter the measured gCNR value, possibly resulting in different classifications of a mass near the chosen gCNR threshold. Therefore (after assessments using standard features presented in B-mode, followed by SLSC images, then introducing gCNR when there is uncertainty, as summarized in Fig. 1), we suggest applying the gCNR classification approach to the image corresponding to the most uncertain orientation. This suggestion aligns with the current standard of breast radiologists providing a mass diagnosis based on the worst features across the radial and anti-radial orientations [68].

From a technical perspective, it is promising that real-time and offline imaging implementations produced similar results when gCNR was applied to SLSC images, despite known differences in signal processing. The 0.73 gCNR threshold was previously chosen based on a linear support vector machine model trained to differentiate solid from fluid masses in offline R-SLSC images [64]. However, other methods are available to determine appropriate thresholds, particularly methods that rely on ROC curves [66,69]. For example, an alternative approach that finds the gCNR threshold closest to (0,1) on the gCNR ROC curve yielded greater and equivalent sensitivity and specificity, respectively, when compared with that achieved with the pre-determined threshold, as reported in Table 1.

One study limitation is that readers have access to multiple sources of information in the clinic, which is assumed to be partially responsible for the generally better performance with real-time (rather than offline) B-mode and SLSC images (reported in Table 1 and shown in Fig. 4). However, in both real-time and offline cases, readers had more contextual information than that available to gCNR when making classifications, yet gCNR can outperform readers when determining fluid or solid content due to its objectivity. While it may be considered a limitation that readers were presented with real-time cases prior to performing the offline reader study, at least 103−124 days elapsed between the real-time and offline readings per radiologist, and the radiologists reported no specific recollection of the imaging findings given the time lapse. Hence, the overlap of cases is not expected to influence the reported performance.

It may also be seen as a limitation that the radiologists included in our study had prior experience reading R-SLSC images [36] and previous exposure to SLSC images [34−36,43,48,50,59,67,70]. Although we were unable to include radiologists with no exposure to SLSC images due to the required training and associated research objectives, the resulting performance reported in Table 1 nonetheless speaks to the ability to learn how to appropriately incorporate SLSC images. In addition, gCNR can potentially provide an alternative training approach for novice radiologists to reduce reader uncertainty with SLSC images.

Future work will evaluate clinically relevant gCNR thresholds with access to larger patient numbers (based on the costs of different clinical decisions), assess reader performance and interactions when presented with quantitative gCNR outputs, and explore automated ROI selections enabled by artificial intelligence. While lowering the gCNR threshold could possibly improve sensitivity (Table 1), doing so may increase the misclassification of malignant solid masses as cysts (which has more detrimental effects when weighed against the benefits of increasing the sensitivity of cyst identification). It is promising that malignant solid masses were correctly classified with the gCNR thresholds of 0.62 and 0.73 applied to real-time SLSC images (Fig. 5a, Fig. 6 and Table 1). While gCNR was assessed outside of the examination room in real-time and offline images in our study, a future analysis of radiologist performance when presented with real-time, objective gCNR classifications will provide additional insight into potential logistical implementations of the proposed clinical workflow. Finally, artificial intelligence can potentially be used to automate ROI selection with no manual ROI selection required, possibly with assistance from multitask deep neural networks that enable simultaneous image formation and segmentation [71,72].

## Conclusion

We successfully evaluated the effectiveness of real-time GPU SLSC imaging relative to offline SLSC imaging when distinguishing complicated cysts from solid hypoechoic breast masses. Applying gCNR to real-time and offline SLSC images yielded AUC values of 0.963 and 0.998, respectively, which are both excellent values with respect to diagnostic testing. Real-time SLSC imaging reduced uncertainty about fluid masses that existed when reading B-mode images alone, thus maintaining this benefit that was previously observed with offline SLSC images. Implementing gCNR as an objective metric when uncertainty remains with SLSC images underscores its potential to enhance the sensitivity and specificity of cystic versus solid differentiation and overcome reader variability and uncertainty. These benefits are promising to reduce 2-year follow-up recommendations (*i.e*., BI-RADS 3 diagnoses) or recommendations for more invasive procedures (*e.g*., biopsy, aspiration).

## Figures and Tables

**Table 1:** Sensitivity and specificity for complicated cyst detection in B-mode and short-lag spatial coherence (SLSC) images, aggregated among the three readers, compared with that achieved with a generalized contrast-to-noise ratio (gCNR) = 0.73 and with the gCNR threshold closest to (0,1) on the real-time SLSC receiver operating characteristic curve (*i.e*., gCNR = 0.62).

	Real-time		Offline	
	Sensitivity	Specificity	Sensitivity	Specificity
**B-mode**				
Readers 1–3	0.67	0.70	0.67	0.55
**SLSC**				
Readers 1–3	1.00	0.58	0.83	0.48
gCNR = 0.73	0.67	0.95	0.67	1.00
gCNR = 0.62	1.00	0.95	1.00	0.95

gCNR, generalized contrast-to-noise ratio; SLSC, short-lag spatial coherence.

## Data Availability

Data from this study will be available upon reasonable request to the corresponding author.
